# Linear Geometric Analysis of Maxillary Expansion in Mixed Dentition: Rapid Palatal Expander Versus Invisalign First System

**DOI:** 10.3390/dj13110504

**Published:** 2025-11-03

**Authors:** Francesca Gaffuri, Francesca Zara, Laura Grassi, Cinzia Maspero

**Affiliations:** 1 Department of Biomedical Surgical and Dental Sciences, University of Milan, 20122 Milan, Italy; francesca.gaffuri@unimi.it (F.G.); cinzia.maspero@unimi.it (C.M.); 2 Fondazione IRCCS Ca’ Granda, Ospedale Maggiore Policlinico, 20122 Milan, Italy; 3 Department of Biomedical and Dental Sciences, Morphological and Functional Images, School of Dentistry, University of Messina, Policlinico G. Martino, Via Consolare Valeria, 98100 Messina, Italy; laura.grassi@studenti.unime.it

**Keywords:** Invisalign First, hyrax, palatal expansion

## Abstract

**Objectives**: This study aimed to evaluate maxillary arch width increase in juvenile patients requiring space gain, but without skeletal transverse discrepancies necessitating orthopedic expansion. The comparison focused on the effects of Rapid Maxillary Expansion (RME) using Hyrax expanders and dentoalveolar expansion via Invisalign First. **Methods**: This retrospective longitudinal study analyzed digital dental models of 38 patients (19 males and 19 females, aged 8 ± 2 years) undergoing maxillary expansion at the Department of Biomedical, Surgical, and Dental Science, Fondazione IRCCS Ca’ Granda, Ospedale Maggiore Policlinico (Milan, Italy). Patients were divided into two groups: one treated with Hyrax expanders (*n* = 19) and the other with Invisalign First (*n* = 19). Intraoral scans were taken before (T0) and after treatment (T1), and measurements were performed using Mimics Materialize 21.0 software. Statistical analysis included *t*-tests, ANOVA, and regression models to assess differences in maxillary expansion between groups. **Results**: Both groups showed statistically significant transverse arch increases (*p* < 0.01). Hyrax achieved greater expansion at the deciduous canine level, while Invisalign showed more at the deciduous molar level. First permanent molar expansion was similar. ICC for reliability was excellent (>0.97). No significant differences in sex or Angle class distribution were observed. **Conclusions**: Hyrax and Invisalign First both produce measurable maxillary expansion, but they serve distinct roles. While Hyrax expanders provide rapid skeletal expansion, Invisalign First offers a less invasive alternative for dentoalveolar widening with controlled force application. They should not be used interchangeably. Appliance selection must be tailored to the severity and nature of the transverse deficiency.

## 1. Introduction

Transverse maxillary deficiency is a prevalent orthodontic issue in pediatric patients, especially during the early mixed dentition phase, with a reported prevalence ranging from 4% to 30% [1]. Its etiopathogenesis is multifactorial, therefore, identifying the causal factors might be difficult. Congenital, functional alterations or traumatic phenomena might be responsible for a decreased development of the upper maxilla leading to a maxillary hypoplasia [2,3]. The palatal expansion is the primary goal for the orthopedic-orthodontic therapy and permit to correct the maxillary transverse deficiencies to increase the upper airways volume and reduce respiratory resistances [4]. Tooth-borne maxillary expansion could be rapid or slow depending on the appliance and the activation protocol [5,6]. From a biological point of view, rapid maxillary expansion (RME) creates large orthopedic forces at the sutural site over a short period and produces immediate mid-palatal suture separation by disrupting the sutural connective tissue [7,8,9]. On the other hand, slow maxillary expansion (SME) occurs with more intermittent and lower forces distributed over longer periods and producing the so-called dentoalveolar expansion. SME creates less tissue resistance around the circum-maxillary structures, with more bone formation in the inter maxillary suture, reducing the force-related side effects of RME [10,11]. Leaf Expander, Quad helix, and Palatal expander are fixed orthodontic appliances currently in use for the SME method [12,13,14,15]. However, nowadays, an innovative approach introduced by Align Technology (Santa Clara, CA, USA) in 2018 to produce a dentoalveolar expansion is the Invisalign First system, designed for younger patients in the early mixed dentition to perform the first phase of orthodontic treatment, including maxillary and mandibular development and dentoalveolar expansion, correction of midline diastema or anterior dental cross-bite and prevention of mesial molar drift [16,17]. The efficacy of RME and SME has been demonstrated over time. The Invisalign First system was investigated by Levrini et al., in 2021, who investigated the dentoalveolar variations after Invisalign First treatment in growing patients; in the same year, Lione et al. analyzed the successive interdental width increase in Invisalign First treatment as well [16,17,18,19]. However, the effectiveness and indications of aligners for expansion remain under investigation, especially when compared to established orthopedic expanders [20,21]. This lack of information makes the dentoalveolar expansion with aligners one of the greatest challenges in Phase I treatment [22]. To the best of our knowledge, while the biological and clinical effects of RME and SME have been investigated in several studies, few previous studies have examined the degree of dentoalveolar expansion achieved in patients treated with aligners [23,24,25]. This study compares skeletal expansion (Hyrax) to dentoalveolar aligner-driven widening (Invisalign First) and discusses their respective indications, effectiveness, and clinical limitations.

## 2. Materials and Methods

### 2.1. Study Design

This longitudinal retrospective study investigated digital dental models of 38 patients requiring treatment for maxillary expansion at the Department of Biomedical Surgical and Dental Science, Fondazione IRCSS, Ca’ Granda, Ospedale Maggiore Policlinico (Milan, Italy). All records were collected before and after the orthodontic-orthopedic treatment between January 2023 and March 2024. This study was conducted according to the declaration of Helsinki and approved by the Research Protocol of “Fondazione IRCCS Cà Granda Ospedale Maggiore Policlinico”. Operative Unit 420, Current Research N. 1, year 2022.

### 2.2. Sample Selection

The study included 19 males and 19 females between 6 and 10 years old (mean age 8 ± 2 years). All subjects were selected according to the following inclusion criteria: Caucasian ethnicity, mixed dentition with well-preserved deciduous second molars, fully erupted permanent upper first molars, high level of compliance and hygiene, and posterior transverse discrepancy of 4–6 mm. All included patients had no previous orthodontic treatment and informed consent, signed by parents or legal guardians, was obtained. The exclusion criteria were presence of multiple and/or advanced caries, presence of supernumeraries or agenesis, periodontal disease, patients with documented pathologies, poor oral hygiene or myofunctional dysfunction, and patients with obstructive sleep apnea syndrome (OSAS).

### 2.3. Clinical Intervention

The choice of orthodontic appliance was made freely by the legal guardians. After the group allocation based on the parents’ preference, the sample was divided into two groups:

- 19 subjects to treat with Hyrax Expander (Forestadent, Pforzheim, Germany) (ten males and nine females)
- 19 subjects to treat with Invisalign First System (nine males and ten females)

**Figure 1:**
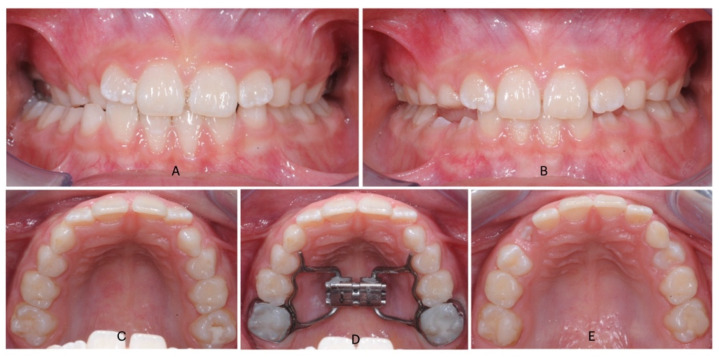
Intraoral photos of Hyrax treatment in a patient with a contracted upper arch and a monolateral cross-bite: (**A**) (pre-treatment frontal view); (**B**) (post-treatment frontal view after overcorrection); (**C**) (pre-treatment occlusal view); (**D**) (occlusal view after cementation of Hyrax); (**E**) (post-treatment occlusal view).

**Figure 2:**
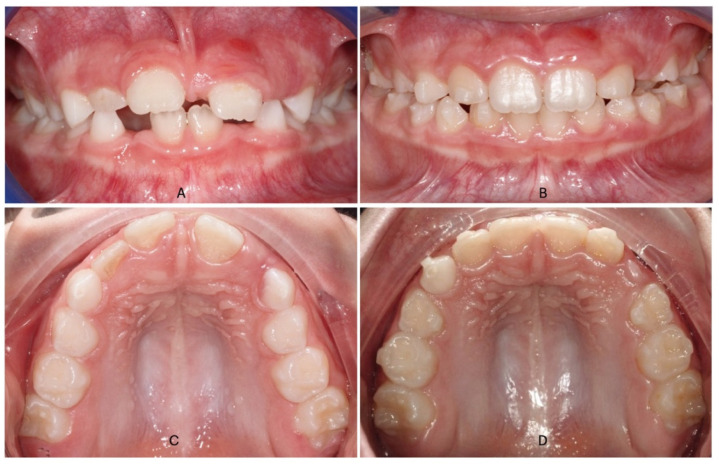
Intraoral photos of Invisalign first treatment in a patient with a contracted upper arch a no cross-bite: (**A**) (pre-treatment frontal view); (**B**) (post-treatment frontal view after Invisalign expansion protocol); (**C**) (pre-treatment occlusal view); (**D**) (post-treatment occlusal view).

Hyrax Expanders had 10 mm central screw anchored on the upper second deciduous molars with fluoride-releasing glass ionomer cement. The expander screw was activated by patients’ parents two times a day (0.25 mm per activation) up to a total of maximum 40 activations (max 10 mm); 4 mm of overcorrection was considered for patients who decided for RME. The entire orthodontic treatment lasted for 10 months after 3 months of active phase and 7 months of stabilization (Figure 1). The second group treated with the Invisalign First system followed a standardized expansion protocol with a carefully planned Clin Check: expansion protocols that began with the molars movement first, followed by the concurrent expansion of primary molars and canines (Figure 2). The amount of arch expansion was 0.15 mm per alignment for a maximum total of 40 aligners (max 6 mm). All patients were instructed to wear full-time aligners with a weekly change and a monthly orthodontic check. Optimized attachments were positioned on incisors, canines, second deciduous molars, and first permanent molars based on needs, to improve tooth movements, and their predictability. The treatment lasted 10 months and all selected patients showed great compliance.

In both groups, patients were properly instructed to follow standard oral hygiene procedures during the treatment period. No adverse reactions were reported in either group, and no patients expressed complaints regarding discomfort or lack of collaboration. These findings suggest that both treatment protocols were well tolerated and did not negatively affect patient compliance or daily oral care.

### 2.4. Clinical and Virtual Measurements

**Figure 3:**
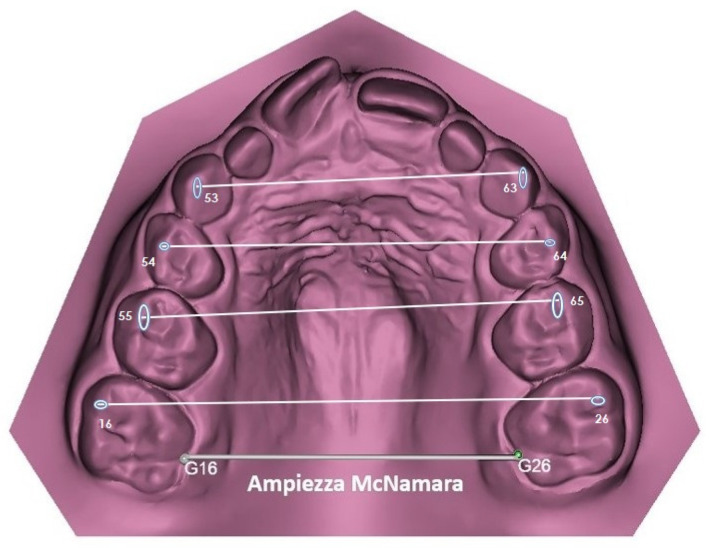
Digital measurements taken from each digital model, before and after orthodontic treatment.

Intraoral scans of the upper and lower arch of all patients were taken before (T0) and after orthodontic treatment (T1) using the iTero scanner (iTero; Align Technology, San Josè, CA, USA). All digital dental models were downloaded as STL files and analyzed with Mimics Materialize 21.0 software (Materialize, Leuven, Belgium). A template consisting of points, lines, and planes of the upper and lower arch of the digital dental model was constructed within the software. The following reference points were identified (Figure 3): vestibular cusps of right and left upper deciduous canines, first and second deciduous molars and first permanent molars. In addition, McNamara amplitude was taken as distance between gingiva margins of palatal grooves of right and left first permanent molars (G16–G26) and compared to the distance in the lower arch between 3.6 and 4.6.

### 2.5. Data Analysis and Statistical Assessment

A previous study by Lione et al. was used to calculate the reproducibility and the sample size, which indicated the need for approximately 30 patients to estimate the intercanine width with a 95% confidence interval (CI), a minimum difference of 1.5 mm, and a standard deviation (SD) of 2.0 mm with a power of 80% [18]. All measurements were performed by the same operator. To assess the method error, the measurements of ten patients were randomly selected and repeated by the first operator and a second operator 15 days later. The intra-class correlation coefficient (ICC) test was adopted to assess intra-operator and inter-operator reliability. Statistical analysis of the collected data was performed using SPSS for Windows software (version 23.0; SPSS, Chicago, IL, USA) and data was analyzed using a descriptive statistic. Shapiro–Wilk test was used to check if data was normally distributed. *t*-test was used to compare initial values of the McNamara amplitude and the 3.6–4.6 discrepancy of the two groups. The paired *t*-test was used to compare the T0 and T1 conditions of each group. Changes in different variables, between T0 and T1, were assessed by Anova analysis. Pairwise comparisons were performed using Tukey’s post hoc test with Bonferroni correction. A linear-regression model was used to assess the correlation between the extent of maxillary expansion between the canine elements, deciduous first molar, deciduous second molar, and permanent first molar. The significance level for both Anova and *t*-test was set (*p*-value < 0.05).

## 3. Results

The ICC values for intra-observer and inter-observer reliability showed high concordance for all volumetric measurements evaluated; the mean (±SD, range) intra-observer and inter-observer ICC values were 0.977 (±0.012, 0.967–0.989) and 0.971 (±0.013, 0.954–0.987), respectively. Overall, the method error was considered negligible. The comparison using the *t*-test showed no significant difference between the values; the two groups had comparable maxillary deficits at T0.

With the Shapiro–Wilk test, it was verified that the collected data had a normal distribution. Table 1 shows the mean values of the McNamara amplitude in the two groups at T0. Both had a reduced maxillary amplitude compared to the normal mean values identified by Spillane and McNamara J [16].

Baseline intergroup comparisons between Hyrax and Invisalign First (T0) were performed (Table 2) and a statistically significant increase was observed in all groups (Table 3 and Table 4). The FIRST group showed similar values to the Hyrax group for the first definitive molars. However, Hyrax group showed a greater increase at the level of the deciduous canines than the FIRST group for no cross-bite and monolateral cross-bite sub-groups. No statistically significant differences were observed in angle class and sex distribution among groups.

## 4. Discussion

The results confirm that while both devices achieve maxillary widening, their mechanisms differ. Hyrax creates skeletal expansion by opening the mid-palatal suture, while Invisalign induces controlled dentoalveolar tipping. As such, they are not clinically interchangeable. As reported by Bucci et al. (2016), maxillary expansion can be achieved using different appliances, activation protocols, and forces [26] The chosen method significantly influences treatment outcomes and their consequences. This study compared two distinct approaches to maxillary expansion in children with transverse maxillary deficiency: the Hyrax rapid maxillary expander (RME) and the Invisalign First^®^ system. Our findings contribute to the existing literature by providing comparative data on dentoalveolar effects and insights into the relative efficacy of these methods in achieving desired arch width increases. Both the RME with Hyrax and the Invisalign First^®^ system effectively increased transverse maxillary dimensions in early mixed dentition, as demonstrated by significant increases in intercanine, intermolar, and other interdental widths across both treatment groups [27]. While both devices showed comparable efficacy at the level of permanent molars, their effects differed in other region of the arch. Specifically, the Hyrax expander produced slightly greater expansion in the deciduous canine region than the Invisalign First^®^ system. Conversely, the Invisalign First^®^ system achieved more pronounced transverse expansion at the primary teeth level than at the first permanent molars. These results agree with findings by Lione et al. [19] who reported that the greatest expansion occurred for upper first deciduous molars, followed by the second deciduous molars and the deciduous canine [19]. Similarly, their study observed greater expansion in the mesial width of the upper first molars compared to their distal and trans palatal widths [17]. Further comparison between Hyrax and the Invisalign First system is supported by Torbaty et al. [28], who noted greater expansion in maxillary intermolar width with the Hyrax system. Additionally, they suggested that Invisalign First may not be a viable option for cases requiring more than 2.5 mm of intermolar expansion. These differences highlight how each method facilitates transverse arch development while targeting specific dentoalveolar regions differently due to variations in device mechanics and force application. This conclusion is consistent with findings by Silvestri-Biavati et al., who demonstrated that while Invisalign First is less effective for a discrepancy greater than 3 mm, it performs well in cases requiring first molar derotation or anterior alignment [29]. Our results similarly indicate expansion for deciduous first molars and improved anterior dental alignment. This effect can be attributed to the aligner’s design, which fully encases the clinical crown of the teeth. Moreover, the digital planning capabilities of Invisalign First allow for reshaping the arch and early alignment of anterior teeth, contrasting with findings by Solano-Mendoza et al. [30]. In REP treatments, where the clinical crown remains uncovered, dental effects are largely limited to the anchor teeth. Comparable observations were reported by Levrini et al. regarding arch changes with Invisalign^®^ First [16]. The Hyrax appliance achieves rapid maxillary expansion through high, intermittent forces applied via a screw mechanism, stimulating skeletal and dental responses. This method separates the mid-palatal suture, promoting direct skeletal widening while also affecting alveolar bone and tooth positions. The significant orthopedic forces ensure consistent skeletal widening, even in mixed dentition. In contrast, Invisalign First employs clear aligners with sequential staging, delivering low, continuous forces that drive gradual dentoalveolar adaptation. This mechanism primarily affects the dentoalveolar complex rather than the mid-palatal suture, enabling controlled dental repositioning with minimal force-related side effects [31,32]. Overall, our findings indicate that both methods achieve similar results in mild to moderate maxillary deficiencies, with Invisalign First serving possible alternative rather than the traditional slow maxillary expansion in cases of mild crowding or limited transverse deficits. Pre- and post-treatment overlays of digital dental models confirmed that clear aligners can provide effective outcomes in specific clinical scenarios.

From a clinical perspective, it is important to interpret these statistical findings in terms of practical thresholds for treatment planning. An increase of ≥2 mm in intercanine or intermolar width is generally regarded as clinically significant to resolve mild crowding or cross-bite. Mild transverse deficiencies (<3 mm) can often be effectively managed with Invisalign First, offering advantages in comfort and esthetics, whereas moderate to severe deficiencies (>3–4 mm) require skeletal expansion with Hyrax to ensure stable correction.

Accordingly, Hyrax should be preferred for patients with marked skeletal discrepancies or when rapid space gain is needed, while Invisalign First is best suited for early interceptive management of mild cases, anterior alignment, and situations where compliance and oral hygiene are favorable. Contraindications for Invisalign include severe skeletal discrepancies and poor compliance, while Hyrax is not indicated in very mild cases where orthopedic expansion would be unnecessary. Defining these thresholds and application scopes provides a more individualized framework for clinicians in selecting the most appropriate appliance.

This study has certain limitations: The retrospective design may introduce potential selection bias, as treatment choice was based on parental preference rather than random assignment. Caregivers may favor aligners-based therapy due to its esthetic and comfort advantages. Additionally, while compliance was high in both groups, differences in adherence to protocols may have influenced the results.

Another crucial consideration is long-term stability. Skeletal expansion achieved with Hyrax is generally supported by sutural remodeling, which may provide more durable retention of transverse correction. The existing literature suggests that relapse is more likely when expansion is primarily dentoalveolar, emphasizing the need for extended retention and careful post-treatment monitoring in aligner-based therapy.

For this reason, future studies with larger sample sizes and randomized controlled designs are needed to better distinguish skeletal from dentoalveolar expansion outcomes. These investigations should also incorporate systematic long-term follow-up to assess stability and relapse patterns between Hyrax and Invisalign First. Only through prospective data collection will it be possible to confirm the durability of treatment results and to establish clear, evidence-based guidelines for retention protocols.

Indeed, our retrospective study restricted the possibility of controlling variables in advance, probably introducing bias or incomplete information. Moreover, retrospective studies do not allow for randomization, which reduces the ability to establish causal relationships. These factors may affect the generalizability of our findings.

## 5. Conclusions

This study demonstrates that both the Hyrax expander and Invisalign First^®^ system significantly improve maxillary arch width in young patients, with specific benefits and limitations associated with each approach. However, RME with Hyrax remains highly effective to gain more space for the anterior teeth because it promotes a greater expansion in the canine region. Conversely, Invisalign First^®^ is a less invasive, aligner-based alternative that promotes gradual dentoalveolar widening with fewer force-related side effects. Despite these differing mechanisms, both approaches successfully address transverse deficiencies in early mixed dentition, providing essential insights for personalized treatment planning in pediatric orthodontics.

## Figures and Tables

**Table 1:** Mean values of maxillary width according to McNamara in the three groups.

T0	No Cross-Bite		Monolateral Cross-Bite		Bilateral Cross-Bite	
	Mean	SD	Mean	SD	Mean	SD
Hyrax Expander	31.68	1.69	31.74	1.87	30.99	1.76
First	31.93	1.75	31.88	1.93	30.48	1.80

**Table 2:** Baseline Intergroup Comparisons Between Hyrax and Invisalign First (T0).

Variable	Hyrax (Mean ± SD)	Invisalign (Mean ± SD)	*p*-Value
**UC**	31.03 ± 1.85	32.30 ± 1.54	0.028
**UD**	37.44 ± 1.69	38.22 ± 2.32	0.244
**UE**	42.51 ± 1.71	43.86 ± 2.37	0.053
**U6(16–26)**	48.43 ± 2.14	49.89 ± 2.40	0.055
**P6**	33.43 ± 1.63	34.87 ± 2.69	0.055
**L6**	44.30 ± 2.29	45.19 ± 1.92	0.206

**Table 3:** Mean differences (Mean) with standard deviation (SD) of the values at T0 and T1.

Interdental Width in Hyrax Group	No Cross-Bite			Monolateral Cros-Bite			Bilateral Cross-Bite		
	T1-T0		*p*-Value	T1-T0		*p*-Value	T1-T0		*p*-Value
	Mean	SD		Mean	SD		Mean	SD	
53–63 (mm)	3.98	2.21	0.002	4.17	1.53	0.002	4.28	1.43	0.001
54–64 (mm)	4.15	1.75	0.001	4.56	1.64	0.001	4.82	1.66	0.001
55–65 (mm)	4.02	2.14	0.002	4.29	2.23	0.003	4.95	1.85	0.002
16–26 (mm)	3.08	1.25	0.001	3.31	1.46	0.001	3.56	1.50	0.004

**Table 4:** Mean differences (Mean) with standard deviation (SD) of the values at T0 and T1 of the FIRST group.

Interdental Width in First Group	No Cross-Bite			Monolateral Cross-Bite			Bilateral Cross-Bite		
	T1-T0		*p*-Value	T1-T0		*p*-Value	T1-T0		*p*-Value
	Mean	SD		Mean	SD		Mean	SD	
53–63 (mm)	3.93	1.73	0.001	4.04	1.81	0.001	4.16	1.79	0.001
54–64 (mm)	4.13	1.75	0.001	4.21	1.69	0.001	4.62	1.66	0.001
55–65 (mm)	4.08	1.84	0.002	4.32	1.73	0.003	4.87	1.65	0.002

## Data Availability

The data that support the findings of this study are available from the corresponding author upon reasonable request.
